# Adenovirus-Mediated eNOS Expression Augments Liver Injury after Ischemia/Reperfusion in Mice

**DOI:** 10.1371/journal.pone.0093304

**Published:** 2014-03-25

**Authors:** Arun P. Palanisamy, Gang Cheng, Alton G. Sutter, John Liu, David N. Lewin, Julie Chao, Kenneth Chavin

**Affiliations:** 1 Division of Transplant Surgery, Department Of Surgery, Medical University of South Carolina, Charleston, South Carolina, United States of America; 2 Pathology and Laboratory Medicine, Medical University of South Carolina, Charleston, South Carolina, United States of America; 3 Biochemistry and Molecular Biology, Medical University of South Carolina, Charleston, South Carolina, United States of America; Rutgers, The State University of New Jersey, United States of America

## Abstract

Hepatic ischemia/reperfusion (l/R) injury continues to be a critical problem. The role of nitric oxide in liver I/R injury is still controversial. This study examines the effect of endothelial nitric oxide synthase (eNOS) over-expression on hepatic function following I/R. Adenovirus expressing human eNOS (Ad-eNOS) was administered by tail vein injection into C57BL/6 mice. Control mice received either adenovirus expressing LacZ or vehicle only. Sixty minutes of total hepatic ischemia was performed 3 days after adenovirus treatment, and mice were sacrificed after 6 or 24 hrs of reperfusion to assess hepatic injury. eNOS over expression caused increased liver injury as evidenced by elevated AST and ALT levels and decreased hepatic ATP content. While necrosis was not pervasive in any group, TUNEL demonstrated significantly increased apoptosis in Ad-eNOS infected livers. Western blotting demonstrated increased levels of protein nitration and upregulation of the pro-apoptotic proteins bax and p53. Our data suggest that over-expression of eNOS is detrimental in the setting of hepatic I/R.

## Introduction

Nitric oxide (NO) is a small, highly diffusible molecule that is synthesized from the amino acid L-arginine by the enzyme NO synthase (NOS). NO is an endothelium-derived vasodilator and is involved in the regulation of systemic blood pressure and local organ blood flow. NO may also modulate reactive oxygen species (ROS) mediated cellular damage, possibly through its inhibitory effects on mitochondrial respiration [1], [2]. Three isoforms of NOS serve different physiological functions as a result of their expression patterns [3]. Neuronal NOS (nNOS) is constitutively expressed in neuronal tissues and regulates neurotransmission, inducible NOS (iNOS) not expressed in most cell types but is highly inducible by bacterial endotoxin and inflammatory cytokines, endothelial NOS (eNOS) is constitutively expressed at low levels in a variety of cells [3]. All NOS isoforms are present in the liver with iNOS and eNOS having wider distribution than nNOS, which is restricted to nerve endings around larger blood vessels [4].

Hepatic ischemia/reperfusion (I/R) injury occurs in a variety of circumstances when liver blood flow is interrupted, including during liver transplantation, liver resection, and shock. Hepatic I/R is a major cause of morbidity and mortality after liver surgery. I/R injury is compounded by multiple factors, including ATP depletion, infiltration/activation of neutrophils/macrophages, formation and release of cytokines, and the production of ROS [5]. However, the mechanisms and mediators involved in I/R injury of the liver remain largely unknown [6], [7]. Some evidence suggest that NO acts as a protective factor during l/R injury. Administration of NO precursors (L-arginine or FK409) into the hepatic vasculature enhanced canine survival following I/R, with increased hepatic blood flow and decreased neutrophil infiltration during the reperfusion period [8], [9]. Similar protective effects were also observed in porcine livers [10], and in murine livers [11], [12] following l/R, while inhibition of the NO production in rats resulted in more severe injury of hepatocytes and endothelial cells [13], [14]. Liver injury is more severe in eNOS-deficient and iNOS deficient mice subjected to liver I/R, compared to wild type counterparts [15).]Likewise, another study demonstrated increased graft injury when eNOS-deficient liver grafts were transplanted into wild type mice. Using an adenoviral vector for human iNOS, over-expressed iNOS in cultured rat hepatocytes resulted in sustained NO production and protection of hepatocytes from spontaneous and TNF-α induced apoptosis [16].

However, considerable controversy exists in understanding the role of NO during I/R of the liver as several studies show NO to have no or detrimental effects on liver I/R. Inhibition of iNOS in pig livers resulted in improved survival rate, decreased levels of serum AST and lactate dehydrogenase, and decreased apoptosis after warm I/R [17], [18]. NO production in the ischemic liver induced *in vivo* by pretreatment with lipopolysaccharide led to increased hepatic injury during reperfusion that was partially ameliorated by the administration of a NOS inhibitor in rats [19], [20]. In another study, NO synthase inhibitor nitro-L-arginine failed to produce any effect on the post-ischemic oxidative stress or neutrophil infiltration in ischemic rat livers [21]. Similar to the liver, attenuation of l/R injury was also observed in the kidneys of iNOS knockout mice [22] and in the kidneys of rats pretreated with iNOS inhibitors [23]. To determine the role of eNOS during hepatic l/R injury we have utilized an adenovirus that over-expresses the human eNOS gene as developed previously by Smith *et al* [24]. We treated mice with Ad-eNOS before I/R and investigated the outcome. In this report, we provide evidence that over expression of eNOS results in increased damage to the liver, with decreased hepatic ATP content and increased apoptosis.

## Materials and Methods

### Ethics Statement

The use of animals is necessary in this study because of the nature of information sought. All rodents used for surgeries were initially anesthetized using isoflurane in desiccators then followed by isoflurane as needed. Animals were observed post-operatively for signs of distress as in respiratory distress, blood pressure, and discernable pain. Buprenorphine was given as an analgesic drug to reduce pain and discomfort. Animals are removed from the study and euthanized by exsanguination (under anesthesia) or CO2 when clearly suffering negates the need to continue humanely in accordance with the Medical University of South Carolina's Institutional Animal Care and Use Committee (IACUC) policy. This study was reviewed and approved by the Medical University of South Carolina's IACUC (AR# 3003: Effects of Steatosis on Ischemia/Reperfusion and Liver Regeneration).

### Animals

Six to eight week-old male inbred C57BL6 lean mice were used in all surgical procedures (Jackson Laboratory, Bar Harbor, ME). Mice were housed in temperature and light controlled chambers on a 12 h:12 h light-dark cycle and provided with water and food adlibitum. All surgical procedures were performed under clean conditions. All animal experiments were reviewed and approved by the university Institutional Animal Care and Use Committee (IACUC).

### Adenovirus injection and warm I/R

Replication-deficient adenovirus expressing eNOS human cDNA under the control of the CMV promoter was made as described previously [24]. High titer Ad-eNOS (10^13^ pfu/ml) was injected to mice through the tail vein, which generally results in up to 70% hepatocyte infection [25], [26]. Similarly, adenovirus expressing LacZ alone (Ad-LacZ) was injected in control mice. Other controls included mice that received vehicle only (DME cell culture medium). Each group had 6 mice. Three days after adenovirus injection, mice were subject to I/R. Mice were given an i.p. dose of heparin (200 units/kg, approximately 6 units in 500 ul saline for each mouse) before l/R to prevent blood coagulation. Isoflurane was administered as a general inhalational anesthetic in all cases. Following mid-line laparotomy, the hilar portal vessels were identified and ischemia was initiated via a pediatric vessel loop ligature for 60 minutes. Reperfusion was subsequently allowed for either 6 or 24 hrs depending on experimental groups. The mice were sacrificed by exsanguination and occipital-cervical subluxation under anesthesia. Part of the liver was fixed in PBS balanced 4% paraformaldehyde, and the remaining lobes were snap-frozen and stored at −80°C.

### SerumAminotransferase

Aspartateaminotransferase(AST)andalanine aminotransferase (ALT) contents were measured using samples of whole blood collected from the portal vein of each mouse under anesthesia during sacrifice as previously described [27]. The blood samples were allowed to clot at room temperature for 15 minutes, centrifuged at 3500 g for 5 minutes at room temperature to collect serum, and AST and ALT measured.

### ATP assay

Cellular ATP contents were analyzed in triplicate using an ATP assay kit from Thermo Lab systems according to the provided protocol with minor modifications [5]. Briefly, frozen aliquots of liver were homogenized in ice-cold RIPA buffer (l50 mM NaCI, 50 mM Tris, 1% Triton X100, 0.1% SDS, 1% deoxycholate, pH 7.5) supplemented with 0.5% trichloroacetic acid just before use. Tissue homogenate was diluted 1∶100 in Tris buffer (0.1 mM, containing sodium acetate). Fifty μl of each diluted sample was mixed with 450 μl reconstituted luciferase solution, and the ATP concentration was measured luminometrically. Total cellular protein from each sample was determined by BCA assay (Pierce, Rockford, IL) for normalization among samples.

### Histology

Paraformaldehyde-fixed paraffin embedded liver sections were stained with hematoxylin-eosin (H&E) for histological analysis [28], and read by a pathologist blinded to the experiment.

### Immunohistochemistry

For immunohistochemical staining, paraformaldehyde-fixed, paraffin-embedded liver sections were used as described previously [27]. Briefly, slides were incubated in polyclonal rabbit anti eNOS antibody (Sigma-Aldrich, St.Louis, MO) 1∶200 dilution in PBS (plus 1% BSA). Slides were washed with PBS and incubated with horseradish peroxidase-conjugated goat anti-rabbit antibody (Pierce, Rockford, IL) 1∶500 dilution for 1 hour and washed with PBS. To localize peroxidase activity, slides were incubated with 3, 3′-diamino-benzidine (DAB) in the presence of H_2_O_2_.

### In-situ detection of apoptosis

TUNEL assay was done on paraformaldehyde-fixed, paraffin embedded liver sections as described previously [29], [30]. Endogenous peroxidase activity was blocked with 2% H_2_O_2_, 5 minutes at room temperature. Liver sections were subsequently incubated with terminal deoxynucleotide transferase (TdT) and digoxigenin-dUTP at 37°C for one hour. The incorporated digoxigenin-dUTP was detected by peroxidase-conjugated anti-digoxigenin antibody and signal developed by incubation with 3, 3′-diamino-benzidine (DAB) in the presence of H_2_O_2_.

### Immunoblotting

Total proteins of the liver were used for Western blot analysis as described previously [5]. Briefly. Liver specimens were homogenized in RIPA buffer containing 5% mammalian proteinase inhibitor (Sigma-Aldrich, St.Louis, MO). Protein concentrations were determined by BCA assay kit from Pierce. 50 ug protein samples were run on 4-12% NuPage polyacrylamide gels (Life Technologies, Grand Island, NY) and transferred to nitrocellulose membranes. After blocking with TBS containing 0.05% Twjecn-20, and 5% milk for 30 minutes, blots were incubated overnight at 4°C with following primary antibodies diluted in TBS-T containing 5% milk: 1) mouse anti nitrotyrosine antibody at 1∶1000 dilution (EMD Millipore, Billerica, CA); 2) rabbit anti eNOS Ab at 1∶10,000 dilution (Sigma-Aldrich, St.Louis, MO); 3) rabbit anti bcl-2 Ab at 1∶1000 dilution (Santa Cruz, Dallas, TX); 4) rabbit anti bax Ab at 1∶1000 dilution (Santa Cruz, Dallas, TX); 5) mouse anti bad Ab at 1∶500 dilution (BD Biosciences, San Jose, CA); 6) mouse anti p53 Ab at 1∶200 dilution (EMD Millipore, Billerica, CA). The blots were washed TBS, incubated with goat anti-rabbit or goat anti-mouse antibodies tagged with peroxidase at 1∶5,000 dilution in TBS containing 5% milk (Pierce, Rockford, IL) for 30 min. After 3×5 min washing with TBST, blots were incubated with freshly prepared Lumi-Light Western Blotting substrate solution (Roche Diagnostics) for 5 min and exposed to X-ray film.

### Statistical analysis

Statistical significance was determined by ANOVA followed by appropriate post-hoc test. A p-value of <0.05 was considered statistically significant. All data are given as mean + standard deviation.

## Results

### Survival and general observation

The activities of each mouse were monitored before and after anesthesia/surgery. All mice survived the I/R insult up to 24 hrs after ischemia. Ad-eNOS injected mice appeared less active after I/R, while mice that received Ad-LacZ behaved similarly to controls. Ad-eNOS animals recovered from anesthesia after approximately 4 hrs, while other groups awoke within 1 hour. Livers from Ad-eNOS injected mice displayed marked congestion and edema, particularly after 24 hrs of reperfusion, a phenomenon that was not observed in other groups.

### eNOS is expressed in Ad-eNOS infected livers

**Figure 1:**
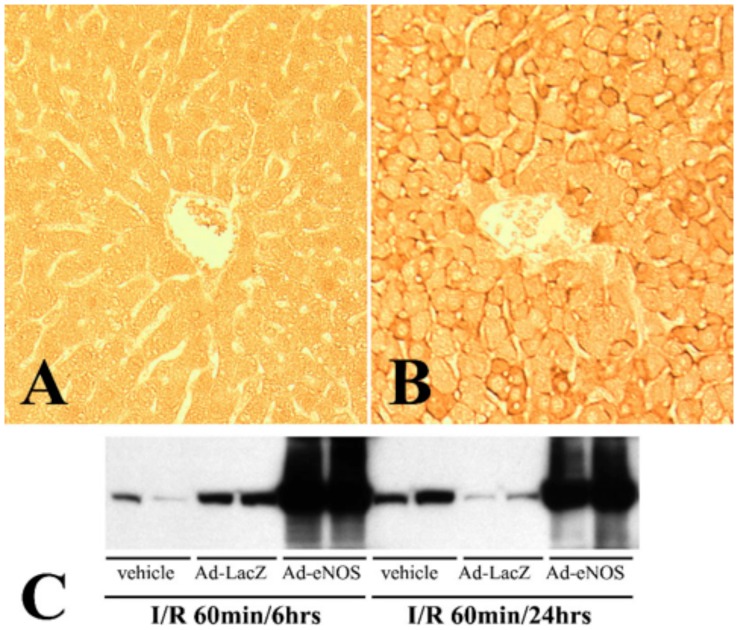
Expression of eNOS in the mouse liver. Immunohistochemical staining of eNOS in mouse liver injected with A) Ad-lacZ, 6 hrs after reperfusion and B) Ad-eNOS, 6 hrs after reperfusion. C) Western blot of eNOS in mouse livers subjected to 60 minutes of ischemia and 6 or 24 hrs of reperfusion as indicated.

To confirm adenoviral infection of the mouse liver, immunohistochemical staining of eNOS in formalin-fixed, paraffin-embed liver sections was performed with rabbit anti-eNOS antibody. eNOS expression in the liver was barely detectable in the control and Ad-LacZ infected groups subjected to either 6 hrs or 24 hrs of reperfusion (Figure 1a). In contrast, in Ad-eNOS groups, a majority of cells in the liver exhibited much stronger eNOS immunostaining, indicating these cells were infected with adenovirus and expressed high levels of eNOS (Figure 1b). The adenovirus-infected cells were evenly distributed throughout the liver and no clusters of strong eNOS immunotstaining were observed. Western blot analysis of total protein with rabbit anti-eNOS antibody was performed to quantify eNOS expression levels in the liver. High levels of eNOS expression were found in Ad-eNOS samples (Figure 1c). Densitometric analysis of Western blots revealed that eNOS expression in the livers of Ad-eNOS injected mice was approximately 20 fold higher than in other groups (Figure 1d).

### Ad-eNOS infected livers have greater AST and ALT, but less ATP after I/R

**Figure 2:**
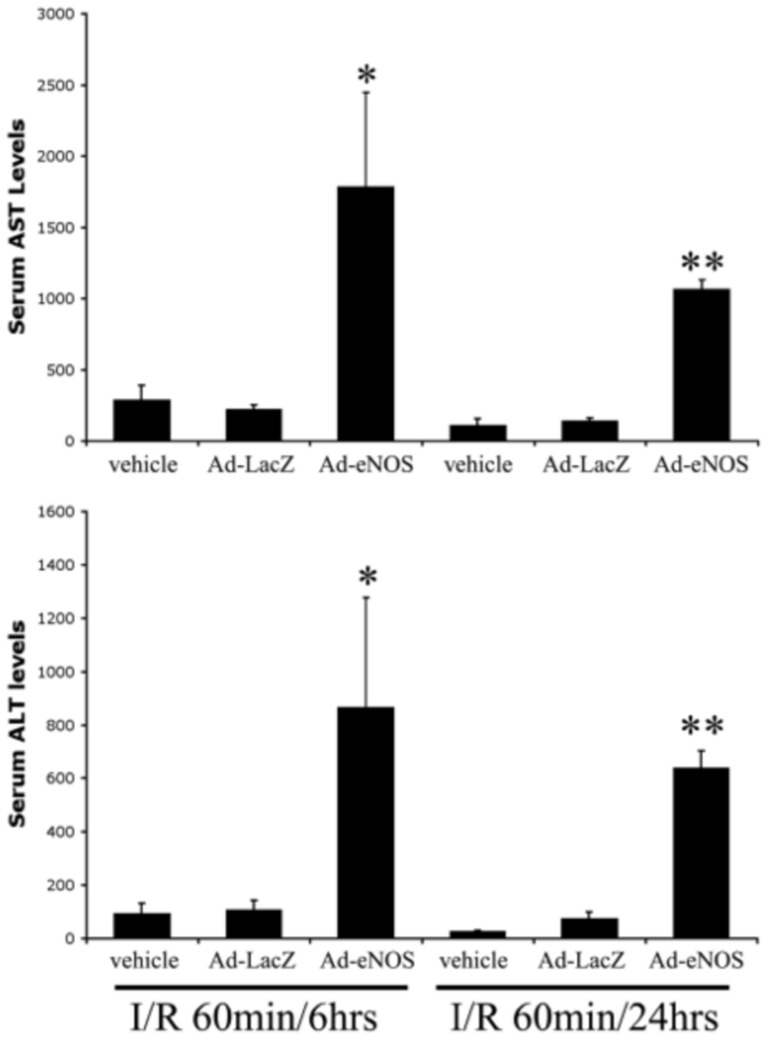
Over-expression of eNOS leads to increased hepatocellular injury as indicated by serum AST/ALT. Mouse livers were subjected to 60 minutes of ischemia and 6 or 24± SD. *p<0.05, **p<0.01 vs. Ad-lacZ injected group.

Serum AST and ALT levels are direct indicators of liver injury. Both AST and ALT levels were dramatically elevated in Ad-eNOS injected mice compared to controls at both 6 and 24 hrs post-reperfusion (Figure 2). Ad-LacZ was not significantly different from non-infected control for either time point (Figure 2).

**Figure 3:**
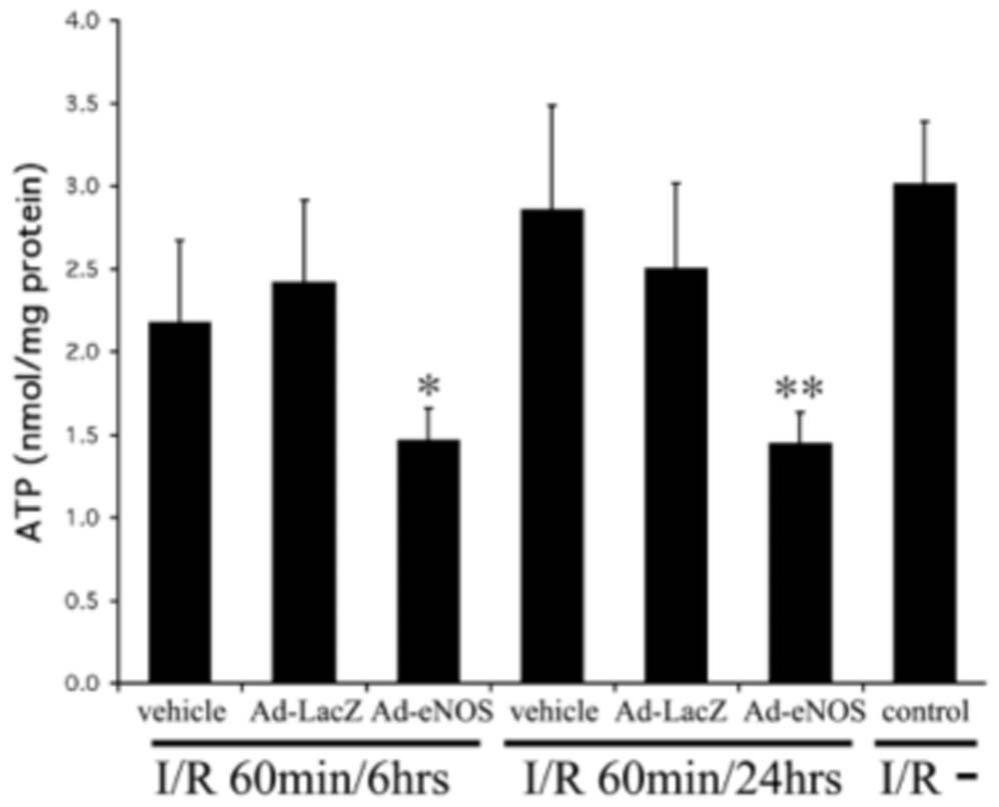
Hepatic ATP content is decreased in eNOS transfected livers after I/R. Mouse livers were subjected to 60 minutes of ischemia and 6 or 24± SD. *p<0.05, **p<0.01 vs. Ad-lacZ injected group.

It has been reported that NO inhibits mitochondrial respiration and de-energizes mitochondria [31]. We evaluated energy state in the livers following I/R by measuring intracellular ATP content via luciferase assay. Ad-eNOS infected livers showed the most significant drop in ATP levels 6 and 24 hrs post-reperfusion (Figure 3). ATP levels in Ad-LacZ infected groups were similar to I/R controls that did not receive adenovirus.

### Ad-eNOS infected livers had increased apoptosis following I/R

**Figure 4:**
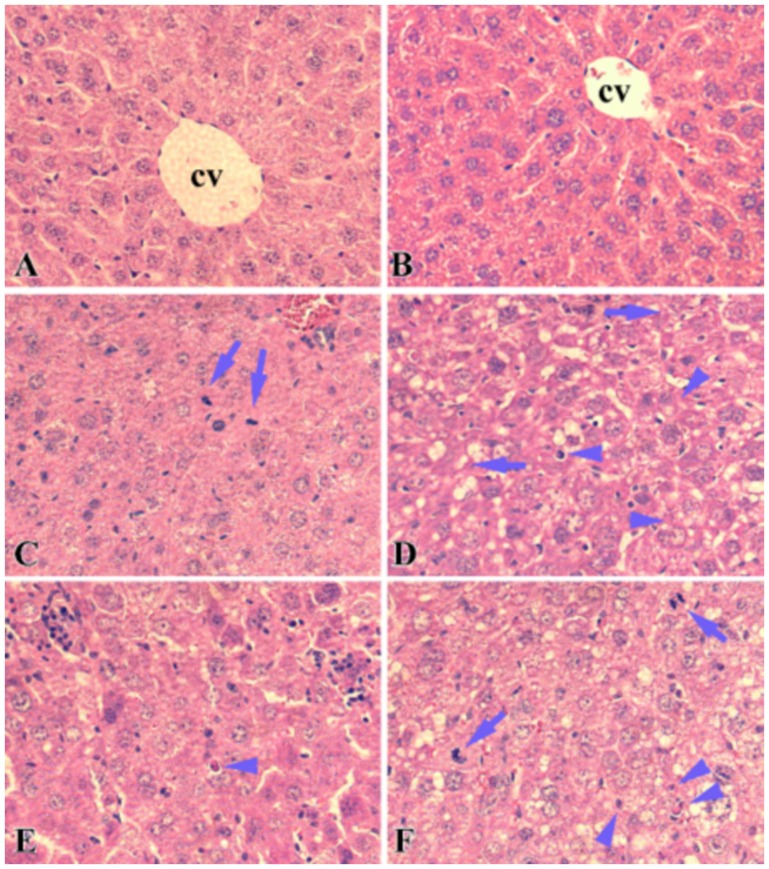
eNOS over-expression leads to fat accumulation and neutrophil infiltration in the liver. H&E staining of the liver sections at 6 (A, C, E) or 24 hrs (B, D, F) of reperfusion. A and B, vehicle control; C and D, lacZ adenovirus infected liver samples; E and F, eNOS adenovirus infected liver samples, cv: central vein; arrow: dividing cells; arrowhead: apoptotic cells.

To understand the underlying pathological mechanism of liver damage in Ad-eNOS infected mice, slides were examined by a clinical pathologist who was blinded to this experiment. Hematoxylin-Eosin (H&E) staining (Figure 4) revealed that, in both Ad-eNOS and Ad-LacZ infected mouse livers, the most remarkable pathological change was fat accumulation in hepatocytes indicating liver steatosis. In vehicle treated livers, the formation of fat droplets was minimal at both 6 and 24 hrs post-reperfusion (Figure 4). However, in both Ad-eNOS and Ad-LacZ infected mouse livers, microvascular (small fat droplets that does not displace the nucleus) and macrovesicular (large fat droplets that displaces the nucleus to the edge of the cell) steatosis, which was detected by 6 hrs after reperfusion, became remarkable with 24 hrs of reperfusion. (Figure 4D, 4F). Infiltration of neutrophils and lymphocytes was also a common feature in livers infected with both adenoviruses (Figure 4C, 4E). Diffuse or focal necrosis was not observed, except for single, isolated, apoptotic cells, which were more commonly seen in Ad-LacZ infected mouse livers (Figure 4E–4F). Mitotic hepatocytes were also commonly observed in both Ad-eNOS and Ad-LacZ infected samples, but not in vehicle controls. However, there were no fundamental pathological differences between Ad-eNOS and Ad-LacZ groups on H&E staining.

**Figure 5:**
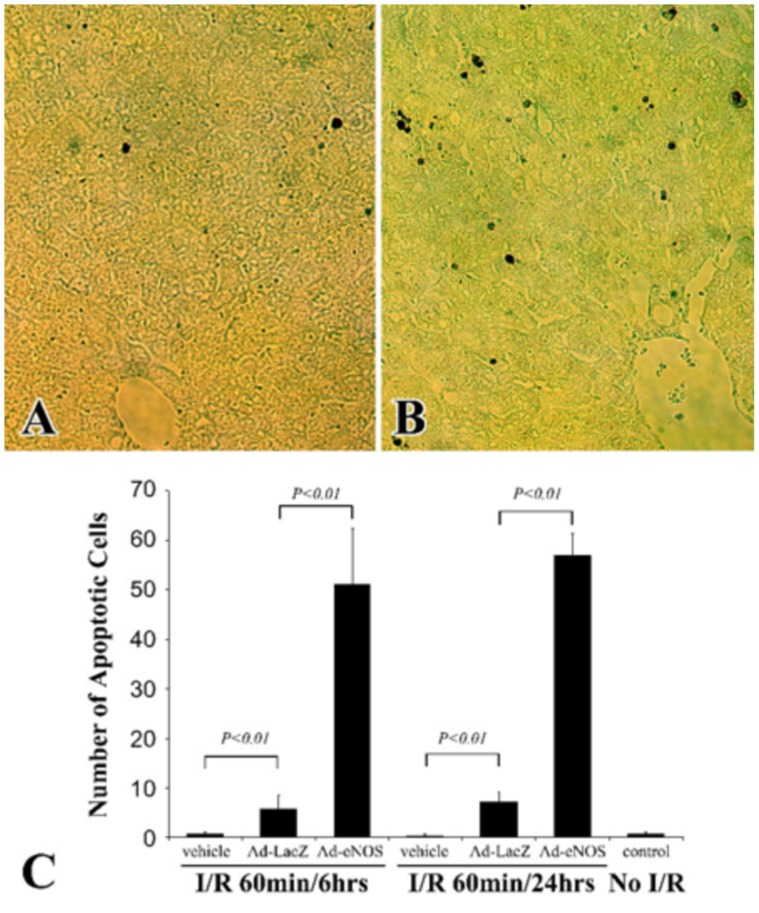
Over-expression of eNOS led to increased apoptotic activity in the liver. A and B, apoptotic cells in lacZ (A) or eNOS (B) adenovirus infected liver samples with 60 min ischemia and 6 hrs of reperfusion, as detected by TUNEL assay. C, the number of TUNEL positive apoptotic cells were counted directly under microscope and expressed as the number of apoptotic cells per high power view. All results are given as mean ± SD.

We next examined whether eNOS overexpression led to increased apoptotic activity in these mouse livers. In-situ detection of apoptotic cells by TUNEL assay revealed that in control livers, there were few hepatocytes undergoing apoptosis after 60 min ischemia and 6 or 24 hrs reperfusion: less than 0.1% of hepatocytes were TUNEL positive. This is consistent with previous reports that apoptotic activity in the ischemic liver is low [32]. Ad-LacZ infection in the mouse liver slightly increased the number of apoptotic hepatocytes (Figure 5A). However, remarkable increase in apoptotic activity was observed in Ad-eNOS infected livers (Figure 5B). Apoptotic cells in Ad-eNOS livers were over 7 and 70 fold more frequent than Ad-LacZ infected and control mice respectively after 6 hrs of reperfusion, and were 20 and 160 fold greater than Ad-LacZ infected and control mice respectively 24 hrs after reperfusion (Figure 5C). In addition, the numbers of apoptotic cells in groups with adenovirus injection remained unchanged from 6 hrs to 24 hrs post-reperfusion, while the control group showed a decrease of apoptosis during this time frame.

### Ad-eNOS infected livers have increased pro-apoptotic proteins bcl-2, bad, p53 and phos-p53 following I/R

**Figure 6:**
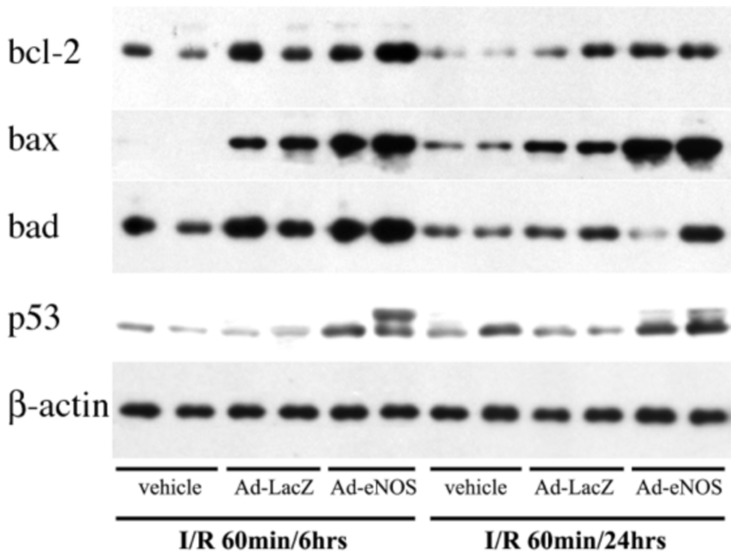
Over-expression of eNOS leads to increased pro-apoptotic proteins in the liver. Western blot analysis of bcl-2, bax, bad, and p53. Total mouse hepatic proteins were used (50 ug of protein per well). The Western blot of β-actin from the same samples was used as an internal control.

To investigate the mechanisms underlying apoptotic changes in Ad-eNOS infected livers undergoing I/R, expression of pro- and anti-apoptotic proteins in total liver protein samples was assessed by Western blot. We found that the expression of bcl-2 and bad was unchanged with LacZ and eNOS adenovirus infection following I/R (Figure 6). However, the expression of bax was increased following I/R in Ad-LacZ infected livers and further increased in Ad-eNOS infected samples. Another pro-apoptotic protein, p53, which had low expression levels in control and Ad-LacZ infected livers, was upregulated in Ad-eNOS infected groups and is also phosphorylated (Figure 6).

### Ad-eNOS infected mice had increased levels of nitrotyrosine following I/R

**Figure 7:**
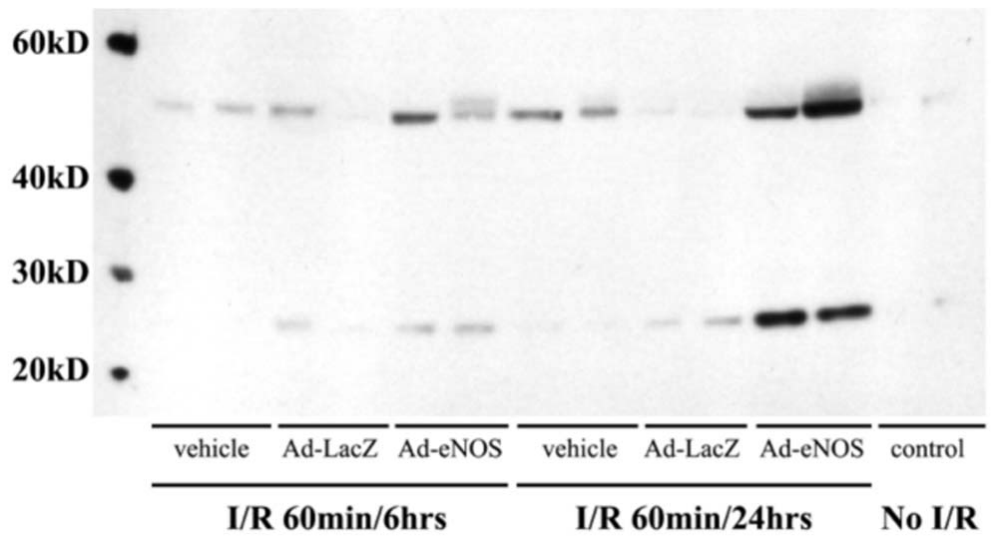
Over-expression of eNOS led to increased levels of nitrosylated proteins in the mouse livers. Two bands (−25 kD and 50 kD) of nitrosylated protein were detected by Western blot analysis of hepatic total proteins.

Peroxynitrite is formed in tissue by NO and superoxide anion [33], and is responsible for tissue damage by NO through nitration of proteins and other cellular components. We examined levels of nitrotyrosine, the footprint of peroxynitrite [34], [35], by Western blot. We found two bands of proteins with nitration, at molecular weights of approximately 25 and 50 kD. Both of bands were stronger in mice that received Ad-eNOS injection (Figure 7). Increased levels of protein nitrosylation were detected by 6 hrs post-reperfusion and became remarkable by 24 hrs.

## Discussion

NO is a free radical endogenously produced in virtually all tissues of the body. It is known to play multiple roles in diverse physiological and pathological processes. This broad functional spectrum includes regulation of vascular tone, immunomodulation, inflammation, inhibition of platelet aggregation, oxidation-reduction, cytoprotection, and apoptosis [36]. Under our experimental conditions, we found that over-expression of eNOS is detrimental to the liver undergoing I/R (Figure 2). This phenomenon was not observed in LacZ adenovirus infected livers, indicating that the augmented damage was caused by eNOS expression and not adenoviral infection. Our data also suggest that the detrimental effect of NO was not only present in the early phase (<6 hrs) of reperfusion damage, but became more prominent during the later phase of reperfusion (6 to 24 hrs). This was evidenced by relatively rapid recovery of AST and ALT at 24 hrs (Figure 2), but slow recovery of ATP (the same level as in 6 hrs) (Figure 3) and increased apoptosis at 24 hrs after reperfusion in Ad-eNOS infected mouse livers (Figure 5).

Several mechanisms may account for I/R-induced liver damage with overloading of eNOS and thus excess NO production. First, insufficient energy supply in the reperfused tissue may be exacerbated by NO excess. Second, since Ad-eNOS infected livers had much lower ATP content than controls following I/R, dysfunction of mitochondria in the liver could be suspected, as mitochondrial dysfunction has been implicated in hepatic I/R injury [5]. A direct effect of NO on tissues is the suppression of energy production by mitochondria. In fact, it is well established that NO and its derivatives inhibit mitochondrial function at various levels. NO potently and reversibly decreases mitochondrial transmembrane potential and inhibits mitochondrial respiration and ATP synthesis in a concentration-dependent manner [31], [37].Even short exposure of rat hepatocytes to NO leads to inhibition of mitochondrial aconitase, NADH-ubiquinone oxidoreductase, and succinate-ubiquinone oxidoreductase [38]. In our experiments, we observed both increased levels of nitrosylated proteins and decreased hepatic ATP content in Ad-eNOS infected mouse livers, strong evidence indicating that the destructive effect of eNOS over-expression are at least partly mediated by suppression of ATP production by NO excess.

Other studies suggest that, in addition to direct toxicity, many of the detrimental effects of NO may be mediated by the formation of peroxynitrite (ONOO^−^) [39], [40]. During I/R, tissue secretes reactive oxygen/nitrogen species including nitric oxide (NO), superoxide (O_2_), and hydrogen peroxide (H_2_O_2_), which alone or in combination may damage the function of hepatocytes [41]. NO (and other superoxide anions) react quickly and spontaneously to form peroxynitrite, a potent and versatile oxidant which affects many signal transduction pathways [33], [35]. The formation of peroxynitrite may cause the irreversible inhibition of complexes I-III-dependent mitochondrial oxygen consumption and significant inhibition of the activities of succinate dehydrogenase and ATPase, leading to inactivation of electron transport components in living cells and tissues [42], [43] and thus potentiation of I/R injury. In addition, the peroxynitrite anion causes lipid peroxidation, DNA breakage, and protein modification including nitration of tyrosine or tryptophane residues, all of which can result in loss of cellular viability [44].

NO also contributes to apoptosis via triggering of the mitochondrial permeability transition, generation of ROS, induction of tyrosine nitration of proteins, and activation of caspase-3 [45]–[48]. Inhibition of iNOS results in improved survival and decreased apoptosis and necrosis after I/R in the pig liver [17], [18]. Similar effects of NO on apoptosis have been observed in the brain [49] and intestinal tract [50]. This evidence is consistent with our finding that Ad-eNOS infected livers had much higher level of apoptosis than the controls following I/R. However, it is not clear how NO and peroxynitrite trigger the apoptotic machinery. Mitochondria are the bioenergetic and metabolic centers of a cell and play a central role during the initiation of apoptosis [51]. Thus, the detrimental effects of NO/peroxynitrite on normal mitochondrial function suggest that dysfunction of mitochondria may underlie the association between eNOS over-expression and apoptosis after I/R. In our experiments, we found that some pro-apoptotic genes (bax and p53) were upregulated in Ad-eNOS infected mice, while expression of the anti-apoptotic gene bcl-2 was not changed. Bax is a pro-apoptotic member of the bcl-2 protein family that resides in the outer mitochondrial membrane. An increased level of bax modulates endoplasmic reticular and mitochondrial calcium stores[52], and interrupts the physiological balance of bax versus other anti-apoptotic genes (e.g., bcl-2) [53], both of which are critical to execution of apoptosis. More important, bax directly induces release of cytochrome c from mitochondria into the cytoplasm, a process that promotes the assembly of a multiprotein complex that induces proteolytic processing and activation of caspases [54], [55]. In addition, the initiation of apoptosis in Ad-eNOS infected livers could also be due to DNA damage caused by NO and peroxynitrite, which may directly lead to programmed cell death or upregulation of p53, another pro-apoptotic gene. Upregulation of p53 is commonly seen in conditions of NO excess [56], [57] and causes growth arrest and induces apoptosis [58]. The upregulation of pro-apoptotic genes, together with the effects of ATP depletion and dysfunction of mitochondria induced by NO and peroxynitrite, all contribute to prosecution of apoptosis and loss of hepatic function during I/R.

In summary, we observed no protective effect with increased expression of eNOS in the mouse liver undergoing I/R injury. Rather, our data provide new evidence that over-expression of eNOS aggravates tissue damage during hepatic I/R.
